# Disease versus disease: Paolo Zacchia on syphilis and epilepsy

**DOI:** 10.1177/09677720221129856

**Published:** 2022-12-13

**Authors:** Jacalyn Duffin, Daryn Lehoux

**Affiliations:** 1 4257 Queen's University, Kingston, ON, Canada; 2 98617 Queen's University Faculty of Arts and Science, Kingston, ON, Canada

**Keywords:** Paolo Zacchia, epilepsy, syphilis, miracles, pathocoenosis

## Abstract

The lawyer and physician Paolo Zacchia (1584–1659) was the chief physician at the Vatican and an important advisor to the papal court. He is considered a founder of the field of forensic pathology, and the influence of his masterwork, *Quaestiones medico-legales*, spread throughout Europe. In this essay, we focus on one of Zacchia's consultations, first published posthumously in 1661. Emerging from a cause for beatification, the case features the intriguing medical notion of one disease curing another. Zacchia was to determine if a young man's recovery from epilepsy was miraculous or not. We will briefly review Zacchia's career, examine his argument and the sources on which he based his reasoning in this case, trace the status of the disease-versus-disease notion to the present, and demonstrate that this consultation represents a rare, if not the only example of syphilis being the curative agent – rather than the disease cured.

Trained by Jesuits in both law and medicine, Paolo Zacchia (1584–1659) served as chief physician in the Vatican and as advisor to the papal court during the reign of two popes: Innocent X and Alexander VII.^1,2^ A prominent consultant and educator, Zacchia was predeceased by his spouse, Terrenzia Cossi, and left no descendents. His will is preserved in the Archivio di Stato, Rome.^3^ Although he may have had Jewish origins, he was a devoted Christian, and his funeral was a grand affair in the Chiesa Nuova (Santa Maria in Vallicella) of Rome.^4^ He published three books. Two were short works in Italian: *Il vitto quaresimale* (1637) a disquisition on Lenten lifestyle, and *De’ mali hipochonrdriaci* (1639), analysis of a physical not mental disease. The third book, begun in 1621 and growing in further editions, was his master work in Latin, *Quaestiones medico-legales* (also *Quaestionum medicolegalium)* (Figure 1)*.* Zacchia's great influence in forensic medicine has attracted scholarly attention, especially in Germany and Italy, where he is viewed as a founding “father.”^5–9^ The journal of the Italian society for forensic pathology bears his name, *Zacchia: Archivio di medicina legale, sociale, e criminologica*. Partly because most of his work remains untranslated and possibly because he served the Roman Catholic church, Zacchia is less well known in the English-speaking medical world. Only recently has the anglophone literature begun to take stock of his significance, situating his life and portions of his work in the broader context of early modern medicine, religion, and jurisprudence.^10–17^

**Figure 1.**
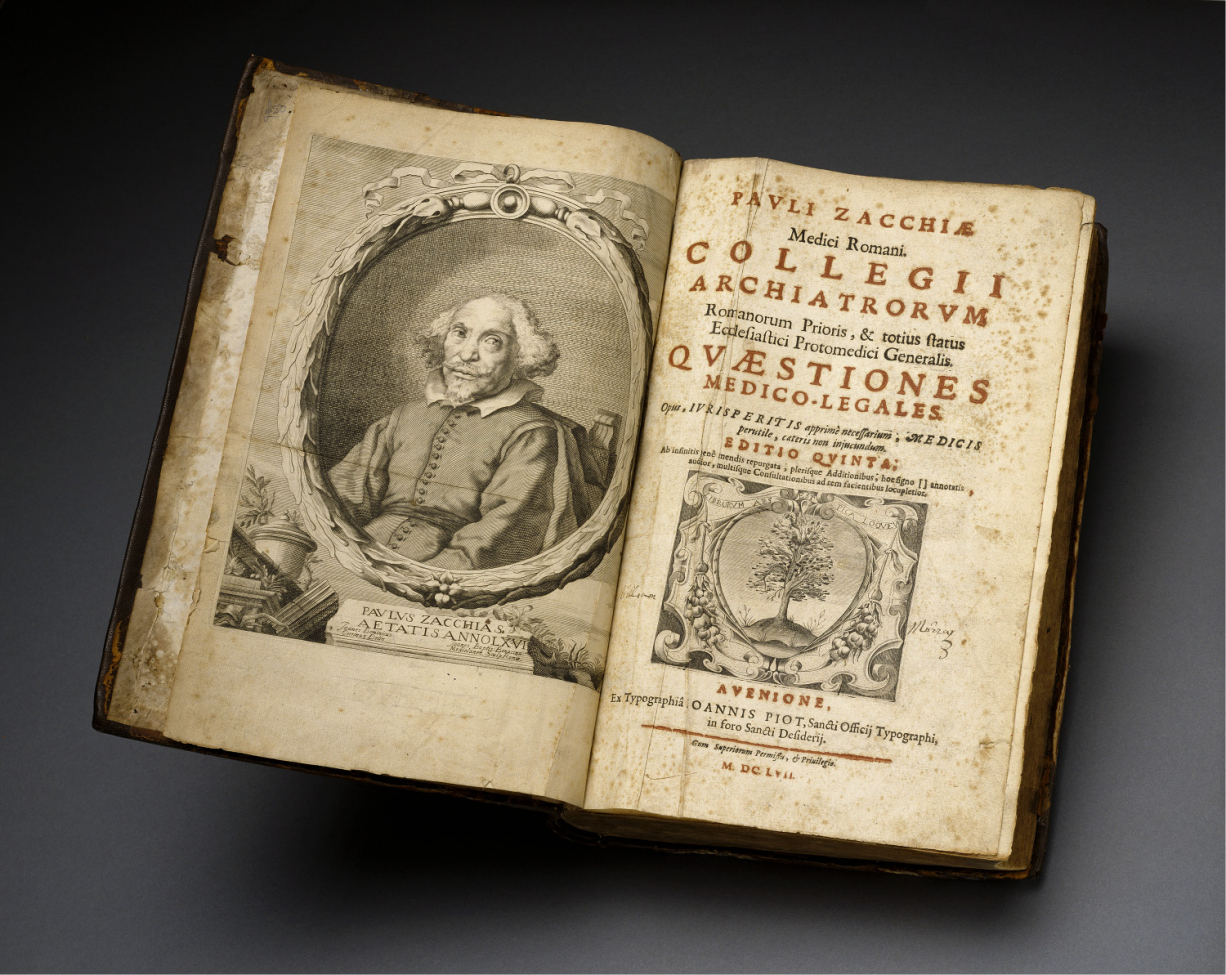
Frontispiece portrait of Paolo Zacchia and title page of the 1657 Avignon edition of his *Quaestiones medico-legales*. Courtesy National Library of Medicine, Bethdesda, MD.

Zacchia's monumental treatise on forensic medicine, *Quaestiones medico-legales*, appeared in successive editions during and after his life from 1621 to 1789. By 1661, it contained 85 consilia (consultations), each one illustrating applications of wisdom contained elsewhere in the book. Like case studies in today's medical literature, consilia represent individual patient histories, in which the theoretical, practical, and clinical observations of authors could be applied, contemplated, debated, and tested. With roots in antiquity, they pervaded scholarly writing from the thirteenth to the sixteenth centuries. Several distinguished historians have developed a robust and still growing literature on their origin, forms, and uses in both the law^18^ and medicine.^19–23^ In his study of Giambattista Morgagni, Saul Jarcho observed that investigating an author's consilia could open a window on a much larger work by demonstrating the theory in action.^24^

Zacchia's consilia address various medico-legal questions of health, death, homicide, paternity, inheritance, and sexuality; fifteen consilia concern possible miracles worked by candidates for sainthood. Some of his consilia have been examined, collectively or as individual case studies, by historians.^25–37^ In them, Zacchia cited more than 250 authors from antiquity to the seventeenth century and he included numerous cross references to other parts of the treatise.^38^ In 2008, we began an online, open-access project to translate all the consilia into English, hoping that they might raise awareness of this little-known physician and enable greater access to his *Quaestiones*. At the time of writing, 61 of the 85 consilia have been either translated or adopted for future translation; 24 are still looking for volunteers.^39^

In this essay, we focus on Zacchia's *Consilium LXXX,* first published posthumously in 1661. Emerging from an anonymous cause for beatification, it features the intriguing medical notion of one disease curing another. Zacchia was consulted to determine if a boy's recovery from epilepsy had been a miracle, or not. We will examine the sources on which Zacchia based his reasoning, trace the status of this idea today, and demonstrate that this consilium represents a rare, if not the only example of syphilis being the curative agent – rather than the disease cured.

That a systemic condition might *prevent* or protect a person from another disease has considerable currency today. The best-known example is the protection offered by prior infection with cowpox virus (vaccina) against smallpox (variola), the dairy maid's observation exploited by Edward Jenner (1749–1823), which led to vaccination and the eventual eradication of smallpox in 1979.^40^ Now, especially in the field of genetics, a relative evolutionary advantage against infectious pathogens has been invoked to explain the persistence of nefarious alleles that, in the homozygous state, can cause serious disease. Thus, for example, we have evidence that two blood disorders—sickle hemoglobin and thalassemia—protect against malaria, or that the iron storage condition, hemochromatosis, protects against plague.^41,42^

By contrast, observations that a new disease can not only prevent but *reverse and even cure* another already present have become rare. Nevertheless, as we will show, such reports date back to antiquity and were transmitted for centuries. The closest analogy in our time may be the observation that the metabolic changes owing to pregnancy (which is not a disease!) can sometimes favorably affect chronic conditions, such as arthritis.^43^

## Zacchia's *Consilium LXXX*: a summary

In mid-seventeenth-century Italy, a boy, named Michael, suffered from periodic seizures. They began at age seven, followed phases of the moon, grew worse with overeating, and steadily increased in severity. When the lad was fifteen, his desperate family consulted a holy man who instructed him to fast, to say a Hail Mary, and then to eat an apple, which the man had blessed. The boy did as he was told. But at around the same time, he had “dallied with a maidservant” and contracted a severe dose of the “French pox.” Large swellings in his groin opened and oozed pus for a lengthy period. Nevertheless, following both the religious rituals and the venereal disease, the boy was free of his seizures “for many years.” After the holy man died, his devotees presented this story as a miracle in the cause for his beatification. The lawyer-physician Paolo Zacchia was asked to examine the case. He eventually decided that the recovery was natural—not a miracle. He supplied a variety of reasons, but relied, in particular, on the ancient notion that one disease can cure another. He wrote, “For it is a common experience that one disease is a remedy for another.”^44^

Who was the would-be saint? Miracles are needed for beatification or canonization. Whenever Zacchia refuted the miraculous nature of a cure, he did not identify the candidate for sainthood, knowing full well that other more convincing cases might be brought forward. He indicated that the “same man” had appeared in another consilium, in which he was said to have prayed successfully, “while he was alive” at an unspecified date, for the miraculous revival of a boy thought to have drowned in an unspecified river–a recovery that Zacchia also rejected as a miracle.^45^ In that consilium, reference was made to the 1656 plague in Rome. The fact of the beatification process, which cannot proceed before a candidate's death, and the phrase, “while he was alive” in the present consilium, imply that the man had died before 1659 when Zacchia himself died and certainly by 1661 when the case was published. This chronological window suggests five seventeenth-century holy men who were not beatified (or canonized) until later: Robert Bellarmine (1542–1621 beat. 1923; can. 1930), Pietro Casani (1572–1647, beat. 1995), Joseph Calasanz (1557–1648, beat. 1748; can. 1767), Vincent de Paul (1581–1660, beat. 1731; can. 1737) and Andrea Bobola (1591–1657 beat. 1853; can. 1938). Among men who have been beatified or declared venerable, no other candidates stand out. For location in Rome and reputation for miracles, the most plausible candidates seem to be Casani or Calasanz; however, many causes for beatification are begun without ever resulting in success. The holy man's identity must remain a mystery.

## Zacchia's argument and sources

Then as now, producing evidence of a natural cure was sufficient to refute the possibility of a miracle in the canonization process.^46^ Zacchia made no comment as to whether or not Michael's venereal disease had been treated medically; however, elsewhere in his consilia, he cited Girolamo Fracastoro (1478–1553) and the treatment of syphilis with mercury and guaiacum.^47^

To refute the miraculous aspects of Michael's recovery, Zacchia set up several plausible arguments in favour of a natural, durable cure. He offered three reasons why the seizures might not have been incurable. First, the boy had reached puberty—an age when spontaneous remissions might occur, and the trigger had been external from overeating, which the lad had curbed having previously struggled to do so. Second, the fever could have removed it by a sort of coction or heating. And third, the oozing buboes could have, by copious evacuation of “rotting humours,” cleansed the “malignant humours stimulating epilepsy.” Zacchia concluded: “Therefore this disease could be overcome by means of medical—which is to say *natural*—assistance and by nature itself, not requiring a divine power intervening specially for its removal. And so vanishes every hint of a miracle; for when nature works by itself, the effect is never to be reckoned as miraculous, as is plain, because a miracle is an effect independent of a natural cause, either in itself or in the chain of events.”^48^

Zacchia's works display astonishing erudition. Some editions of his *Quaestiones* provide a list of more than one thousand authors cited within.^49^ In his consultation on Michael's putative miracle, Zacchia cited several ancient authors. Hippocrates and Aretaeus were invoked to establish the difficulty of curing epilepsy once it had become chronic, although they had observed that some sufferers grew out of it spontaneously at puberty.^50^ Zacchia also cited several passages from Galen on the curative power of quartan fever (a form of what is now malaria). In the *Commentary on Epidemics 1,* 3.4, Galen cited Hippocrates’ *Epidemics* 1.24: “the quartan … also fends off other serious diseases.”^51^ He also wrote, “we see epilepsies stopping when quartans are attacking for a long time, but you know already about how this happens from what is written in *On the Differences of Fevers* and *On Crises*.”^52^ It is interesting that in this section, Galen parsed the Hippocratic passage about “other serious diseases” (νοσημάτων ἑτέρων μεγάλων) simply as “other diseases” (καὶ ἄλλων νοσημάτων), although he carried on immediately to refer to epilepsy specifically. When Galen discussed a similar passage in *Epidemics 6*, he explained that “the serious disease” is a technical term for epilepsy specifically.^53^ Wesley D. Smith's translation of the *Epidemics 6* gives this same passage as “People seized with quartan fevers are not seized with epilepsy. If they have it already and a quartan fever supervenes, they are cured.”^54^

Zacchia also cited Avicenna's *Canon*, where it is written, “one disease is the treatment for another, such as a quartan, by which sometimes epilepsy is cured, and gout, and varices, and pain of the joints” (trans. Lehoux).^55^ Other uncited sources lend credence to Zacchia's claim that the beneficial effects of quartan fever were well known—a “common experience.” The popular 1560 *vademecum* of Jodocus Lommius, *Observationes medicinales*, which appeared in many editions and translations into the late eighteenth century, included a similar passage: “Persons labouring under a Quartan [fever] are neither afflicted with Madness, nor Melancholy, nor a Leprosy, nor Convulsions arising from Repletion. Besides, if these Disorders have previously rag’d, they are generally terminated upon the approach of a Quartan. Some Physicians also assert that no Person can die of a Quartan, except by his own or his Physician’s Fault.”^56^ The last line came, like so much of Lommius, directly from Celsus.^57,58^

## Beyond Zacchia

We tend to think of diseases as immutable entities. In fact, they are also elaborate ideas about suffering—concepts—to explain symptoms, and they are conditioned by science and culture. Although histories are usually written about single diseases, physician-historian Mirko Drazen Grmek invented the neologism, *pathocoenosis*, to describe the constellation of diseases prevalent in a specific time and place.^59,60^ He wrote, “Two diseases belonging to a single pathocoenosis can be in a state of symbiosis, antagonism, or indifference to each other.”^61^ In *Consilium LXXX*, Zacchia can be said to have discussed a sub-population-level pathocoenosis involving two diseases: one ancient and one relatively new. Invoking many venerable authors, he asserted that antagonism between diseases was capable of prevention or cure.

The ancient notion that one disease could cure another persisted for at least two more centuries beyond Zacchia. It may have slipped into the general view of the healing power of nature.^62^ It is found in the works of the early nineteenth-century French alienist, Philippe Pinel (1745–1826). He wrote that unnamed “ancient and modern physicians” described cures of inveterate insanity by various other diseases, including “jaundice, phlegmonic eruptions, varicose swellings, hemorrhoidal evacuations, by quartan fever, etc.”^63^ Pinel later went on to describe three cases of insanity cured by other diseases, two of which he had witnessed personally and one drawn from the sixteenth-century author, François Valleriola (1504–1580).^64,65^ Pinel also cited Zacchia on the difficulty of curing complicated insanity.^66^ In this context, it is important to remember that epilepsy was considered to be a disorder of the mind, akin to, if not a form of mental illness. Indeed, Pinel classified epilepsy as a variety of *alienation mentale*, along with hypochondria, mania, melancholia, sleepwalking, rabies, tetanus, and muscular weakness.^67^

In 1841, surgeon and apothecary William James West (1794–1848) of Tonbridge, Kent, wrote to the *Lancet* to describe seven months of convulsions in his infant son that had resolved following an acute fever. He was convinced that the initial ailment was a previously unrecognized disease *sui generis*, the rare type of infantile spasms now called West syndrome.^68,69^

Only with the work of the English neurologist John Hughlings Jackson (1835–1911), in the 1860’s and 1870’s, was epilepsy given a firm anatomical basis as a neurological disorder and gradually teased away from psychiatric conditions to become an organic disease of the brain with distinctive manifestations.^70–72^ It is perhaps significant that the late nineteenth-century colonies for epileptics still tended to be run by psychiatrists.^73–75^ Nevertheless, the newly appreciated anatomical nature of epilepsy enhanced interest in physical treatments.

William P. Spratling (1863–1915), director of the Craig Colony for Epileptics in New York state, reviewed the research that had been conducted from 1899 to 1902 on epilepsy and concurrent diseases. Recognizing that Hippocrates had described benefits with malaria (quartan fever), he found that his own experience revealed only a few instances of mild improvement following several infections, including malaria, erysipelas, measles, and typhoid. Spratling also noted that, rather than helping, sometimes the febrile illness triggered status epilepticus. Given this evidence, researchers agreed that “the chances for improvement are too uncertain to warrant the utilization of bacterial products in the practical therapeutics of epilepsy, as has been proposed.”^76–78^

The separation of mental illness and epilepsy would be consolidated with the advent of electroencephalography in the 1920’s and effective drugs, phenobarbital (1918) and phenytoin (Dilantin, 1938).^79^ Indeed, epilepsy could be a *cause* of mental distress and illness, but it was no longer a mental illness. Therefore, the apparent leap by psychiatrist Julius Wagner-Jauregg (1857–1940) to the use of fever—and malaria in particular (although he used the tertian form not quartan)—in treating an organic disease of the mind seems less jarring: it emerged from well-established tradition. For this research, which began in 1888, he was awarded the Nobel Prize in 1927.^80–82^ Wagner-Jauregg himself cited ancient sources that had recognized the benefits of periodic fever against epilepsy and psychosis, and he relied on his own clinical observations.^83^ He experimented with other fever-inducing toxins, such as erysipelas and tuberculin, but by 1917, he settled on the malaria parasite because it could be easily eliminated with treatment after allowing a few cycles of periodic fever. The method became standard therapy for neurosyphilis in many centers until it fell into disuse with the advent of penicillin in the 1940s. Cohort studies have confirmed that Wagner-Jauregg's fever therapy “worked.” For example, patients treated over a three-decade period between 1924 and 1954 at the Vincent Van Gogh Institute for Psychiatry, in Venray, Netherlands, tolerated the therapy well and lived longer than others.^84^

The success of Wagner-Jauregg's method and other documented recoveries following various fevers led to considerable fascination with generalized “pyrotherapy,” on one hand, and therapeutic infections, on the other. We find traces of both today. As recently as 2016, when a case of psychosis improved following bacteremia, discussants speculated on the mechanism of action, recognizing that the fever and/or the antibiotics and/or the infection could have been responsible for the cure.^85^

Reports of naturally occurring fever provoking cures of epilepsy have recently emerged from China,^86^ Italy,^87^ and Japan.^88–90^ They describe temporary and permanent remissions of West syndrome and other childhood seizures following infections with high fevers in clusters of up to 25 patients. The authors insist upon the striking coincidence in timing, but caution against the logical fallacy of *post hoc ergo propter hoc*, because—as Zacchia had observed—youths can outgrow seizures. They also remark on the paradoxical nature of the observation, as convulsions are far more frequently *provoked* by high fevers rather than cured. The “curative” pathogens were proven or presumed to be viruses, including varicella (chicken pox) and rotavirus (gastroenteritis). While these observations have not resulted in trials to deliberately generate fever for managing epilepsy, they foreshadow the recent use of localized sterile heat against epilepsy: MRI-guided, induced hyperthermia, or laser interstitial thermal therapy (LITT), which deliver temperatures of around 43°C to precise areas of the brain.^91,92^

Examples of deliberate use of infectious agents to “treat” other diseases abound. The dubious legend that tapeworm diet pills were widely marketed for weight loss may well be overblown.^93^ But the more euphemistic “helminthic therapy” is now being investigated in clinical trials for immune-modulation in managing allergic and autoimmune conditions, based on the observation that regions of the world with high parasitic infection rates display low incidences of those diseases.^94,95^ Similarly, fecal microbiota transplantation to restore the normal gut flora, first proposed about thirty years ago, has become more prominent owing to the rising incidence of *Clostridium difficile* infection.^96^ Granted the bacteria used in the procedure are not supposed to be pathogenic and normally do not produce disease, although recent reports warn that it is a possibility.^97^

Nevertheless, syphilis seems not to have been used in that way, although it has been deliberately inoculated for experimental purposes rather than cure.^98^ These experiments begin with a famous inoculation by John Hunter (1728–1793), possibly leading to his own or another's death.^99,100^ They continue through the misguided attempts to “prevent” syphilis with inoculation of wetnurses by Joseph-Alexandre Auzias-Turenne (1812–1870) and of prostitutes by Albert Neisser (1855–1916); far from immunizing the subjects, the inoculations caused the disease and sent the researchers to court.^101,102^ Vulnerable female prisoners were injected with syphilis by Japanese authorities at Unit 731 in Manchuria and by Nazi doctors at Ravensbrück concentration camp; they sought information about the disease for protecting their own troops or creating bioweapons.^103,104^ Prisoners were similarly used in the United States, and an American study, conducted between 1946 and 1948, entailed deliberately infecting 700 otherwise healthy Guatemalan men and women with syphilis—not to heal them of any other condition—but with the stated goal of assessing the effectiveness of penicillin as a treatment; at least 83 people died.^105^ Since the 1950s, whenever epilepsy and syphilis are mentioned together in current medical literature, it is usually to portray the former as a consequence of the latter.

## Conclusion

In the pathocoenosis of the COVID-19 pandemic, disease-on-disease antagonism has become familiar, usually to emphasize how a pre-existing condition, such as diabetes, obesity, cardiorespiratory disease, or immunocompromise, increases vulnerability to the SARS-CoV-2 virus. Aside from the preventative conditions in evolutionary genetics, statements regarding how one disease might cure another are rare.

Paolo Zacchia's *Consilium LXXX* exposes the vitality and durability of what was once a “well-known” idea that one disease can indeed cure another, especially epilepsy, and it invited us to trace that history into our own time. What is distinctive—if not unique—in Zacchia's analysis of Michael's case is that it was *syphilis,* acting in the place of traditional quartan fever, that might have effected the cure of his seizures. As far as we can determine, this may be the only example of a cure ascribed to naturally occurring venereal disease. It also reminds us of the exquisite observational powers of our predecessors for noticing phenomena that continue to be relevant to human physiology and medicine, even if they are no longer allowed to occur. The advent of specific treatments for control of quartan fever (late seventeenth century, purified in 1944), epilepsy (twentieth century), and syphilis (mid-twentieth century) has led to what Ray and Schulman have called a “Pavlovian treatment response”: all diseases are treated when diagnosed. They observe that the situation has engendered a debate over whether or not untreated fever might actually be beneficial.^106^ The result is that it has become rare to allow *any* disease to run its natural course, let alone two. Therefore, it is no longer “a common experience” to observe how one disease might cure another.

## References

[1] De Renzi S and Tonetti L. Zacchia, Paolo. In: *Dizionario biografico degli Italiani, volume C*. Roma: Istituto della Enciclopedia Italiana fondata da Giovanni Treccani, 2020, pp. 344– 348, https://www.treccani.it/enciclopedia/paolo-zacchia_(Dizionario-Biografico).

[2] Poutrain I . Nouvelles recherches sur la poétesse Debora Ascarelli: juifs, chrétiens et convertis dans la Rome de Clément VIII. Mélanges de l’Ecole française de Rome 2018; 130: 245– 260.

[3] Zacchias P . Archivio di Stato (S. Ivo), Notaio Antonio Franciscus Maria Simi, Cura del Vicario, Officio 32, Rome, March 1659, pp.662–665v and 683–684v.

[4] Zacchias P. Archivio di Stato (S. Ivo), Notaio Antonio Franciscus Maria Simi, Cura del Vicario, Officio 32, Rome, March 1659, p.663.

[5] Pastore A Rossi G (eds) Paolo Zacchia: alle origini della medicina legale 1584–1659. Milan: FrancoAngeli, 2008.

[6] Händel K . Paolo Zacchia–der geistige Vater der Rechtsmedizin [Paolo Zacchia–the spiritual father of forensic medicine]. Archiv für Kriminologie 2003; 212: 65– 73. 14639809

[7] Traunfellner Z . Paolo Zacchia–Vater der Gerichtlichen Medizin, 400 Jahre nach seiner Geburt [Paolo Zacchia–the father of forensic medicine, 400 years after his birth]. Zeitschrift für Rechtsmedizin. Journal of Legal Medicine 1985; 94: 159– 163. 3890416 10.1007/BF00198685

[8] Fischer-Homberger E . Medizin vor Gericht: Gerichtsmedizin von der Renaissance bis zur Aufklarung . Bern/Stuttgart/Wien: Hans Huber 1983: 120– 122, 193–198, 338–341.

[9] Pomata G . Contracting a cure: patients, healers, and the law in early modern Bologna. Roy R Taraboletti-Segre A (trans). Baltimore and London: Johns Hopkins University Press, 1998, pp.35, 46, 61, 87, 112, 118, 138, 149, 159, 167. 15072043

[10] Bouley BA . Pious postmortems: anatomy, sanctity, and the Catholic Church in early modern Europe. Philadelphia: University of Pennsylvania Press, 2017.

[11] De Renzi S . ‘A fountain for the thirsty’ and a bank for the pope: charity, conflicts, and medical careers at the hospital of Santo Spirito in seventeenth-century Rome. In: Grell OP Cunningham A Arrizabalaga J (eds) Health care and poor relief in counter-reformation Europe. London and New York: Routledge, 1999, pp. 102– 131.

[12] Mohr JC . Doctors and the law: medical jurisprudence in nineteenth-century America. Baltimore: Johns Hopkins University Press, 1996, p. 17.

[13] De Ceglia FP . The body of evidence: corpses and proofs in early modern European medicine. Leiden: Brill, 2020.

[14] Gentilcore D . Healers and healing in early modern Italy. Manchester: Manchester University Press, 1998, pp. 23, 24, 186, 187, 194.

[15] Gentilcore D . Medical charlatanism in early modern Italy. Oxford and New York: Oxford University Press, 2006, p. 99.

[16] Maclean I . Logic, signs, and nature in the Renaissance: the case of learned medicine. Cambridge: Cambridge University Press, 2002, p. 87.

[17] Duffin J . Medical miracles: doctors, saints, and healing in the modern world. New York: Oxford University Press, 2009, pp. 21– 25.

[18] Maclean I . Interpretation and meaning in the Renaissance. Cambridge: Cambridge University Press, 1992, p. 30.

[19] Agrimi J Crisciani C . Les consilia médicaux. Viola C (trans). Turnhout, Belgium: Brepols, 1994.

[20] Siraisi NG . Taddeo Alderotti and his pupils: two generations of Italian medical learning. Princeton, NJ: Princeton University Press, 1981, pp. 270– 302.

[21] Siraisi NG . Medieval and early Renaissance medicine: an introduction to knowledge and practice. Chicago and London: University of Chicago Press, 1990, pp. 73, 118, 119, 148, 173, 175.

[22] Crisciani C . Medicine as queen: the consilia of Bartolomeo da Montagnana. In: Manning G Klestinec C (eds) Professors, physicians and practices in the history of medicine: essays in honor of Nancy Siraisi. Cham, Switzerland: Springer, 2017, pp. 79– 96.

[23] Quaranta A . The consilia by learned physicians Pietro Andrea Mattioli and Francesco Partini: dialectic relations between doctrine, empirical knowledge and use of the senses in sixteenth-century Europe. Social History of Medicine 2022; 35: 20– 48. 35264901 10.1093/shm/hkab118 PMC8902007

[24] Jarcho S . The clinical consultations of Giambattista Morgagni. Boston: Countway Library, 1984, p. xxiii.

[25] Jarcho S. *The clinical consultations of Giambattista Morgagni*. Boston: Countway Library, 1984, pp.lxxxi-ii, 96–99.

[26] Fischer-Homberger, *Medizin vor Gericht*, pp.683–687.

[27] De Renzi S . La natura in tribunale: conoscenze e pratiche medico-legali a Roma nel XVII secolo. Quaderni Storici 2001; 108: 799– 822. 18841616

[28] De Renzi S . Witnesses of the body: medico-legal cases in seventeenth-century Rome. Studies in History and Philosophy of Science 2002; 33: 219– 242. 12240683 10.1016/s0039-3681(02)00005-5

[29] De Renzi S . Medical expertise, bodies, and the law in early modern courts. Isis 2007; 98: 315– 322.

[30] De Renzi S . The risks of childbirth: physicians, finance, and women’s deaths in the law courts of seventeenth-century Rome. Bulletin of the History of Medicine 2010; 84: 549– 577. 21196603 10.1353/bhm.2010.0026 PMC3034399

[31] De Ceglia FP . The woman who gave birth to a dog. Monstrosity and bestiality in *Quaestiones medico-legales* by Paolo Zacchia. Medicina nei Secoli 2014; 26: 117– 144. 25702383

[32] Laverda A . Tracing the boundaries of the natural: medicine and the inquiry on miracles in early modern canonization trials. Journal of the History of Medicine and Allied Sciences 2019; 74: 369– 390. 31592528 10.1093/jhmas/jrz053 PMC6906214

[33] Ditchfield S. Liturgy, sanctity, and history in tridentine Italy; Pietro Maria Campi and the preservation of the particular. Cambridge: Cambridge University Press, 2002, p. 235.

[34] Pedrazza Gorlero C . La ferita incriminante: due consilia de vulneribus di Paolo Zacchia (1584–1659). In: Pastore A Rossi G (eds) Paolo Zacchia. Milan: FrancoAngeli, 2008, pp. 200– 220.

[35] Pastore A . Casi di venefici tra cinque e seicento: teoria medicolegale e pratica-penale. In Pastore A Rossi G (eds) Paolo Zacchia. Milan: FrancoAngeli, 2008, pp. 249– 265.

[36] Marchisello A. “Culpa habet sociam poenam”: la responsibilità del medico nelle *Quaestiones medico-legales* di Paolo Zacchia. In Pastore A Rossi G (eds) Paolo Zacchia. Milan: FrancoAngeli, 2008, pp. 221– 248.

[37] Gigliola M Villata R . Paolo Zacchia, la medicina come sapere globale e la ‘sfida’ al diritto. In Pastore A Rossi G (eds) Paolo Zacchia. Milan: FrancoAngeli, 2008, pp. 9– 49.

[38] Duffin J . Questioning medicine in seventeenth-century Rome: the consultations of Paolo Zacchia. Canadian Bulletin of Medical History 2011; 28: 149– 170. 21595366 10.3138/cbmh.28.1.149

[39] The collaborative translation project of the consilia of Paolo Zacchia, https://jacalynduffin.ca/zacchia/ (2008, accessed 1 September 2022).

[40] Bennett B . War against smallpox: Edward Jenner and the global spread of vaccination. Cambridge: Cambridge University Press, 2020.

[41] Goheen MM Campino S Cerami C . The role of the red blood cell in host defence against falciparum malaria: an expanding repertoire of evolutionary alterations. British Journal of Haematology 2017; 179: 543– 556. 28832963 10.1111/bjh.14886 PMC5922454

[42] Moalem S Percy ME Kruck TPA , et al. Epidemic pathogenic selection: an explanation for hereditary hemochromatosis? Medical Hypotheses 2002; 59: 325– 329. 12208162 10.1016/s0306-9877(02)00179-2

[43] Ostensen M Villiger PM . The remission of rheumatoid arthritis during pregnancy. Seminars in Immunopathology 2007; 29: 185– 191. 17621703 10.1007/s00281-007-0072-5

[44] Zacchia P . *Observationum medico-legalium in tres tomos divisae*, volume 3. Lugduni: Anisson and Posuel, 1726, Consilium LXXX [1661], pp.128–129. Translation by Daryn Lehoux at https://jacalynduffin.ca/wp-content/uploads/2018/06/Zacchia-Consilium-80-trans-Lehoux-revised.pdf.

[45] Zacchia P . Observationum medico-legalium, volume 3, Consilium LXXIX [1661], pp.126–128. Translation by Alice Browne at https://jacalynduffin.ca/wp-content/uploads/2018/12/Consilium-79-browne.pdf

[46] Laverda, *Tracing the boundaries*.

[47] Zacchia P . *Observationum medico-legalium*, volume 3, Consilium XXVIII, pp. 44– 45.

[48] Zacchia P. *Observationum medico-legalium*, volume 3, Consilum LXXX [1661], p.129.

[49] The lists appear within front matter of the 1657 and 1688 editions of *Observationum*.

[50] Hippocrates, Sacred disease, ch 14; Aretaeus, Chronic disease lib 1, cap 4.

[51] Kuhn KG . *Galeni Opera Omnia*. Leipzig: Car. Cnoblochi, 1821–1833, 20 volumes (hereinafter, K[volume number]), K17a 226.3–6: ὁ τεταρταῖος … καὶ νοσημάτων ἑτέρων μεγάλων ῥύεται.

[52] K17a 227.10 f.: παυσαμένας γοῦν ἐπιληψίας ἴσμεν ἐπὶ τεταρταίῳ χρονίως ἐνοχλήσαντι, καὶ μεμάθηκας ἤδη περὶ τῆς γενέσεως αὐτοῦ κατά τε τὰ Περὶ διαφορᾶς πυρετῶν ὑπομνήματα καὶ τὰ Περὶ κρίσεων,

[53] K17b 341.

[54] Smith WD (ed) Epidemics 6.6. *In* Hippocrates vol. VII. Loeb classical library 477. Cambridge: Harvard University Press, 1994, p. 249.

[55] Avicenna. *Canon medicinae*, Gerardus (trans), book 1, part 2, thesis 1, ch. 8: est una egritudo alterius egritudinis medicamentum sicut quartana ex qua multotiens sanata epilepsia et podagra et varices et articulorum dolores.

[56] Lommius J. The medicinal observations of Jodocus Lommius. Wynter J (trans). London: W. Owen, 1747, p. 13.

[57] Celsus. A translation of the eight books of Aul. Corn. Celsus on medicine, 2nd ed. Collier GF (trans). London: Simpkin and Marshal, 1831, book 3, chapter 15, p. 98.

[58] Duffin J . Jodocus Lommius’s little golden book and the history of diagnostic semeiology. Journal of the History of Medicine and Allied Sciences 2006; 61: 249– 287. 16606696 10.1093/jhmas/jrj047

[59] Grmek MD . In: Méthot PO (ed and trans) Pathological realities: essays on disease, experiments, and history. New York: Fordham University Press, 2018, pp. 31– 73.

[60] Méthot PO (ed). Médecine, science, histoire. Le legs de Mirko Grmek. Paris: Editions Matériologiques, 2019, pp. 113– 150.

[61] Grmek MD . Diseases in the ancient Greek world. Muellner M and Muellner L (trans). Baltimore: The Johns Hopkins University Press, 1989, p. 3.

[62] Neuburger M . An historical survey of the concept of nature from a medical viewpoint. Isis 1944; 35: 16– 28.

[63] Pinel P . A *Treatise on insanity*, Davis DD (trans), Sheffield: Todd, 1806, p.281. Original French for the translator’s “quartan fever” was the less specific, “fièvres intermittentes.”

[64] Pinel P . Traité medico-philosophique sur l’alienation mentale, 2nd ed. Paris: Brosson, 1809, pp. 379– 384.

[65] Valleriola F. Observationum medicinalium. Lugduni: F. Fabrum, 1605, 2., pp. 140– 166.

[66] Pinel. *Traité medico-philosophique*, p.452.

[67] Pinel. Nosographie philosophique, ou la méthode d’analyse appliquée à la médecine, 3 vols, 2nd ed. Paris: Brosson, pp.1803–1805, tome 3 (1805), Tableau des maladies. np.

[68] West WJ . On a peculiar form of infantile convulsions. Lancet 1841; 35: 724– 725.

[69] Ford JMT . William James West (1794–1848): abdominal surgeon and distraught father. Journal of Medical Biography 2003; 11: 107– 113. 12717540 10.1177/096777200301100213

[70] Swash M . John Hughlings Jackson (1835–1911): An adornment to the London Hospital. Journal of Medical Biography 2015; 23: – 8. 10.1177/0967772013479758 25585567

[71] Critchley M Critchley EA . John Hughlings Jackson: father of British neurology. Oxford: Oxford University Press, 1998.

[72] Jackson JH . Syphilis followed by unilateral convulsions four months afterwards; temporary hemiplegia; paralysis. Medical Times and Gazette 1863; i: 111.

[73] Kissiov D Dewall T Hermann B . The Ohio Hospital for epileptics: the first ‘epilepsy colony’ in America. Epilepsia 2013; 54: 1524– 1534. 24010576 10.1111/epi.12335 PMC3775289

[74] Szasz T . Cruel compassion: psychiatric control of society’s unwanted. Syracuse: Syracuse University Press, 1998, pp. 43– 62.

[75] Lannon SL . Free standing: social control and the sane epileptic, 1850–1950. Archives of Neurology 2002; 59: 1031– 1037. 12056944 10.1001/archneur.59.6.1031

[76] Spratling WP . Epilepsy and its treatment. Philadelphia: Saunders, 1904, pp. 386– 387.

[77] Fine EJ Fine DL Sentz L . The importance of Spratling. Archives of Neurology 1994; 51: 82– 86. 8274114 10.1001/archneur.1994.00540130116019

[78] Fine EJ Fine DL Sentz L , et al. Contributions of the founders of Craig Colony to epileptology and public care of epileptics: 1890–1915. Journal of the History of the Neurosciences 1995; 4: 77– 100. 11619021 10.1080/09647049509525629

[79] Reif PS Strzelczyk A Rosenow F . The history of invasive EEG evaluation in epilepsy patients. Seizure 2016; 41: 191– 195. 27131772 10.1016/j.seizure.2016.04.006

[80] Whitrow M . Wagner Jauregg and fever therapy. Medical History 1990; 34: 294– 310. 2214949 10.1017/s0025727300052431 PMC1036142

[81] Whitrow M . Julius Wagner Jauregg (1857–1940). Journal of Medical Biography 1993; 1: 137– 143. 11615254 10.1177/096777209300100302

[82] Gandevia B . Malaria treatment for general paralysis of the insane [letter]. Journal of Medical Biography 1994; 2: 121.

[83] Wagner Jauregg J . Nobel lecture: the treatment of dementia paralytica by malaria inoculation, delivered 13 December 1927, https://www.nobelprize.org/prizes/medicine/1927/wagner-jauregg/lecture/ (accessed 31 March 2022).

[84] Ouwens IMD Lens CE Fiolet ATL , et al. Malaria fever therapy for general paralysis of the insane: a historical cohort study. European Neurology 2017; 78: 56– 62. 28633136 10.1159/000477900

[85] Zuschlag ZD Lalich CJ Short EB , et al. Pyrotherapy for the treatment of psychosis in the 21st century: a case report and literature review. Journal of Psychiatric Practice 2016; 22: 410– 415. 27648506 10.1097/PRA.0000000000000181

[86] Zeng XD Hu WG . Spontaneous remission of infantile spasms following rotavirus gastroenteritis. Neurological Sciences 2021; 42: 253– 257. 32632632 10.1007/s10072-020-04564-6

[87] Pintaudi M Eisermann MM Ville D , et al. Can fever treat epileptic encephalopathies? Epilepsy Research 2007; 77: 44– 61. 17875384 10.1016/j.eplepsyres.2007.05.012

[88] Hattori H . Spontaneous remission of spasms in West Syndrome: implications of viral infection. Brain & Development 2001; 23: 705– 707. 11701282 10.1016/s0387-7604(01)00278-9

[89] Yamamoto H Yamano T Niijima S , et al. Spontaneous improvement of intractable epileptic seizures following acute viral infections. Brain & Development 2004; 26: 377– 379. 15275699 10.1016/j.braindev.2003.09.012

[90] Fujita Y Imai Y Ishii W , et al. Improvement of intractable childhood epilepsy following acute viral infection. Brain & Development 2011; 33: 62– 68. 20144516 10.1016/j.braindev.2010.01.002

[91] North RY Raskin JS Curry DJ . MRI-guided laser interstitial thermal therapy for epilepsy. Neurosurgery Clinics of North America 2017; 28: 545– 557. 28917283 10.1016/j.nec.2017.06.001

[92] Wicks RT Jermakowicz WJ Jagid JR , et al. Laser interstitial thermal therapy for mesial temporal lobe epilepsy. Neurosurgery 2016; 79: S83– S91. 10.1227/NEU.0000000000001439 27861328

[93] Rance C . Eat! Eat! Eat! Those notorious tapeworm diet pills. *The Quack Doctor*, 23 January 2015, https://thequackdoctor.com/index.php/eat-eat-eat-those-notorious-tapeworm-diet-pills/ (accessed 31 March 2022).

[94] McKay DM . The therapeutic helminth? Trends in Parasitology 2009; 25: 109– 114. 19167926 10.1016/j.pt.2008.11.008

[95] Smith H Forman R Mair I , et al. Interactions of helminths with macrophages: therapeutic potential for inflammatory intestinal disease. Expert Review of Gastroenterology & Hepatology 2018; 12: 997– 1006. 30113218 10.1080/17474124.2018.1505498

[96] Krajicek E Fischer M Allegretti JR , et al. Nuts and bolts of fecal microbiota transplantation. Clinical Gastroenterology and Hepatology 2019; 17: 345– 352. 30268564 10.1016/j.cgh.2018.09.029

[97] DeFilipp Z Bloom PP Soto MT , et al. Drug-resistant E. coli bacteremia transmitted by fecal microbiota transplant. New England Journal of Medicine 2019; 381: 2043– 2050. 31665575 10.1056/NEJMoa1910437

[98] Cuerda-Galindo EX Sierra-Valentí X González-López E , et al. Experimentación en sífilis hasta la Segunda Guerra Mundial: historia y reflexiones éticas. Actas Dermo-Sifiliográficas 2014; 105: 762– 767 [English translation available at the website]. 24268559 10.1016/j.ad.2013.09.007

[99] Gladstein J . Hunter’s chancre: did the surgeon give himself syphilis? [letter]. Clinical Infectious Diseases 2005; 41: 128. 15937780 10.1086/430834

[100] Abdulrahman GO Jr . John Hunter’s (1728–1793) account of venereal diseases. Journal of Medical Biography 2016; 24: 42– 44. 24585621 10.1177/0967772013480701

[101] Sherwood J . Infection of the innocents: wet nurses, infants, and syphilis in France, 1780–1900. Montreal: McGill-Queen’s University Press, 2010.

[102] Benedek TG . ‘Case Neisser’: experimental design, the beginnings of immunology, and informed consent. Perspectives in Biology and Medicine 2014; 57: 249– 267. 25544327 10.1353/pbm.2014.0018

[103] Helm S . This is a woman: inside Ravensbrück, Hitler’s concentration camp for women. London: Little, Brown, 2015.

[104] Gold H Totani Y . Japan's infamous Unit 731: firsthand accounts of Japan's wartime human experimentation program. Tokyo; Rutland, VT; Singapore: Tuttle, 2019.

[105] Reverby SM . Ethical failures and history lessons: the U.S. Public health service research studies in Tuskegee and Guatemala. Public Health Reviews 2012; 34: 1– 17.26236074

[106] Ray JJ Schulman CI . Fever: suppress or let it ride? Journal of Thoracic Disease 2015; 7: E633– E636. 10.3978/j.issn.2072-1439.2015.12.28 PMC4703655 26793378

